# High-Resolution Composition Analysis of an Inactivated Polyvalent Foot-and-Mouth Disease Vaccine

**DOI:** 10.3390/pathogens9010063

**Published:** 2020-01-16

**Authors:** Leonie F. Forth, Dirk Höper, Martin Beer, Michael Eschbaumer

**Affiliations:** Friedrich-Loeffler-Institut, Institute of Diagnostic Virology, 17493 Greifswald-Insel Riems, Germany; leonie.forth@fli.de (L.F.F.); dirk.hoeper@fli.de (D.H.); martin.beer@fli.de (M.B.)

**Keywords:** inactivated vaccine, vaccine matching, composition, strain, deep sequencing, degraded RNA, FMDV, whole genome

## Abstract

Appropriate vaccine selection is crucial in the control of foot-and-mouth disease (FMD). Vaccination can prevent clinical disease and reduces viral shedding, but there is a lack of cross-protection between the seven serotypes and their sublineages, making the selection of an adequately protective vaccine difficult. Since the exact composition of their vaccines is not consistently disclosed by all manufacturers, incompatibility of the strains used for vaccination with regionally circulating strains can cause vaccination campaigns to fail. Here, we present a deep sequencing approach for polyvalent inactivated FMD vaccines that can identify all component strains by their genome sequences. The genomes of all strains of a commercial pentavalent FMD vaccine were de novo assembled and the vaccine composition determined semi-quantitatively. The genome assembly required high stringency parameters to prevent misassemblies caused by conserved regions of the genome shared by related strains. In contrast, reference-guided assembly is only recommended in cases where the number of strains is previously known and appropriate reference sequences are available. The presented approach can be applied not only to any inactivated whole-virus FMD vaccine but also to vaccine quality testing in general and allows for better decision-making for vaccines with an unknown composition.

## 1. Introduction

Foot-and-mouth disease (FMD) is a highly contagious disease that causes severe economic damage, crippling local and global agriculture and commerce. The etiologic agent, foot-and-mouth disease virus (FMDV), infects cloven-hoofed animals, including domesticated and wild ruminants and pigs, leading to acute febrile disease with vesicles in and around the mouth and on the feet [1,2]. Although the mortality rate is low, the morbidity rate can be up to 100% combined with a drastic reduction in the productivity in the infected herds [3,4]. Suitable vaccines can prevent clinical disease and reduce viral shedding, thus limiting the economic consequences, even though subclinical infection and the development of a carrier state can still occur [5,6,7]. In many endemic areas, i.e., parts of Africa, the Middle East, and Asia, FMD control is attempted by regular mass vaccination [8]. 

FMDV is a positive-sense, single-stranded RNA virus in the genus *Aphthovirus*, family Picornaviridae. The FMDV genome is about 8.3 kb in length and encodes one long open reading frame of about 7 kb that is flanked by a long 5’-untranslated region and a shorter 3’-untranslated region, ending with a poly-(A) tail [9]. Like other RNA viruses, FMDV has an error-prone polymerase, leading to a high mutation rate during genome replication and the ability of rapid adaptation [10,11]. The seven serotypes (O, A, C, Asia 1, Southern African Territories 1, 2, and 3) cause indistinguishable disease but are antigenically distinct, with no cross-protection after infection or vaccination [2,12]. Even within a serotype, cross-protection between lineages is variable and the emergence of new variant viruses can lead to a reduced efficacy of existing vaccines [13,14,15]. This antigenic variation constitutes a major problem in the control of FMDV and has to be considered in vaccine development and application.

Conventional FMD vaccines are produced on baby hamster kidney (BHK-21) cell suspension cultures with virulent FMDV, followed by centrifugation to remove cell debris [8]. The virus is chemically inactivated using binary ethylenimine (BEI) and can subsequently be purified by ultrafiltration or PEG precipitation. The use of well-defined purified vaccines that do not contain non-structural viral proteins is an important prerequisite for the differentiation of infected from vaccinated animals [16]. Current FMDV vaccines are formulated with various adjuvants. Depending on the type of adjuvant, the vaccines can be in aqueous or oil form. With aqueous vaccines, aluminum hydroxide gel and saponin may be used as adjuvants while in the case of oil vaccines, single emulsion (water-in-oil) as well as double-oil emulsions (water in oil in water) are available [16]. 

It is of absolute importance to gather up-to-date information on locally circulating virus strains for proper selection of vaccine strains and optimal protection against infection and disease [16]. However, the specific strains used for vaccine production are not always disclosed by the manufacturers [17], leaving insecurities about the expected extent of protection afforded by the applied vaccine against the circulating FMDV strains. Therefore, the possibility of precisely determining the vaccine composition can facilitate the selection of an adequate vaccine. VP1-specific RT-qPCR with subsequent Sanger sequencing has been used for the identification of vaccine strains [18], but this is made difficult by the severe RNA degradation caused by BEI inactivation [19] and possible inhibitory effects from the used adjuvants. With conventional methods, the serotypes contained in the vaccine must be known in order to select suitable primers, and their genetic information must be arduously reconstructed from small fragments. By the open approach of high-throughput sequencing, it is possible to detect the ensemble of genetic material present in a vaccine without any prior knowledge, including not only the different FMDV strains in the case of polyvalent vaccines but also adventitious agents and other contaminants. In this paper, we show that it is feasible to identify and assemble the whole genomes of all strains in a polyvalent-inactivated vaccine by using a workflow based on deep sequencing. It is currently not possible to reliably predict the heterologous protection of vaccine strains based on their genetic sequences alone [20], but once the vaccine composition is known, available in vitro and in vivo data—such as serological relationship coefficients (r_1_-values) and heterologous 50% protective doses (PD_50_)—can be reviewed to decide if the vaccine is suitable for the intended application.

## 2. Results

### 2.1. FMD Vaccines Contain Highly Fragmented Nucleic Acids due to BEI Inactivation

**Figure 1:**
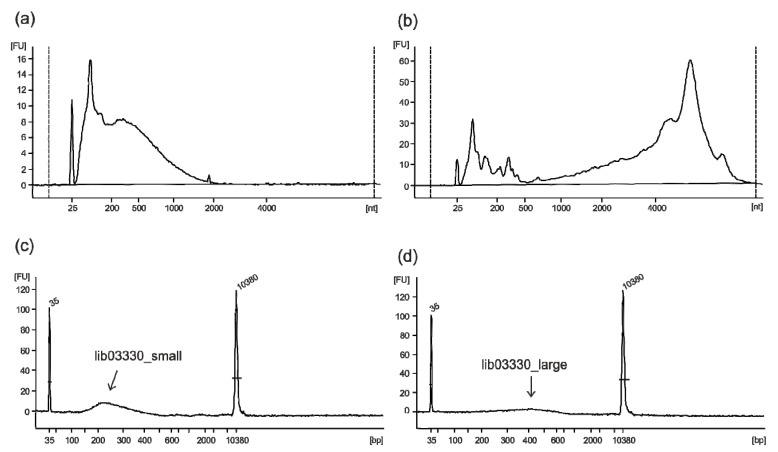
Bioanalyzer results. (**a**) RNA quality of the extracted RNA from the inactivated pentavalent vaccine, showing strong degradation and, for comparison, (**b**) RNA extracted from vesicular fluid containing a large amount of infectious FMDV particles. (**c**,**d**) High Sensitivity chip electropherograms depicting the size distribution of the vaccine library after size selection.

A commercial pentavalent FMD vaccine made from purified virus preparations that had been inactivated by treatment with BEI and formulated with a double oil emulsion (water in oil in water) adjuvant was used for the composition analysis. The extracted RNA (600 ng from 250 µL of vaccine) was highly fragmented, as shown in Figure 1a,b in comparison with RNA extracted from native vesicular fluid containing a large amount of infectious FMDV particles. While the fragmentation of the viral genome during the inactivation process is essential to ensure the safety of the vaccine, it creates a challenging condition for the reconstruction of whole genome sequences by deep sequencing. An adequate library preparation method was chosen to convert the majority of fragments into functional library molecules, aiming in a first step for an insert size of 400 bp. Additionally, in a second step, shorter fragments were collected and retained as backup (lib03330_small). The final libraries covering different insert sizes were quality controlled on a Bioanalyzer HS chip (Figure 1c,d). The library containing the longer molecules (lib03330_large) was sequenced on an Ion Torrent S5 XL, delivering a dataset of 4.2 million reads with a median read length of 277 bp (interquartile range: 106 bp) after trimming.

### 2.2. Vaccine Purity

Using deep sequencing, the genetic material within the sample can be screened for residues from cell culture propagation or other contaminations. A megablast analysis of a data subset of 5000 randomly selected reads against known FMDV genomes identified >99% reads of FMDV origin, demonstrating the high purity of the vaccine. The remaining 23 reads were either too short to obtain a significant match, only distantly related to FMDV, or identified as hamster-derived sequences, very likely residuals of the virus propagation on baby hamster kidney cells (BHK-21). The purity of the vaccine was also supported by a more thorough metagenomic analysis on the complete dataset using the RIEMS software pipeline [21] with 99.2% high-quality reads assigned to FMDV and only a small amount of hamster-derived sequences.

### 2.3. Attempted Identification of Strain Composition Using Megablast

**Figure 2:**
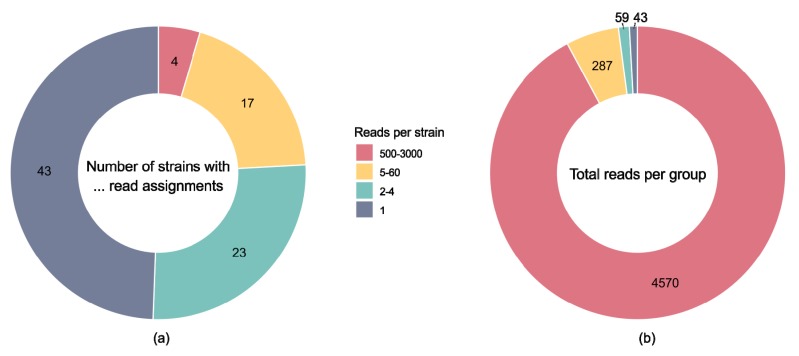
Reads per FMDV strain identified with a megablast search of 5000 reads against all FMDV genomes in the database. (**a**) Matched FMDV strains grouped by number of matched reads per strain. (**b**) Distribution of all FMDV reads across the four groups.

One approach to identify the vaccine strains is by BLAST searches, but this approach requires the genomes of all component strains to be available in the database. Using megablast on a data subset of 5000 reads against known FMDV genomes with every read allowed to match only once, reads were assigned to 87 FMDV strains overall (Figure 2a and Supplementary Table S1). However, 75% of these hits had only one to four matched reads. These matches stem from conserved regions of the FMDV genome leading to reads matching several strains with equal E-value, and, to a lesser extent, from sequencing errors in the reads, but do not indicate the presence of all 87 strains in the vaccine. Of the 5000 reads, the majority (91.4%) were assigned to four virus isolates: FMDV type O isolate “o1manisa iso87” (accession no. AY593823; 2,722 reads), FMDV type A strain “Malaysia 97” (KJ933864; 673 reads), FMDV type Asia 1 isolate “asia1leb-89 iso89” (AY593798; 615 reads), and FMDV type A isolate “a22iraq-95 iso95” (AY593762; 560 reads). Only these four isolates had more than 500 matched reads per strain, and together accounted for 92.2% of the FMDV reads (Figure 2b). The remaining 5.8% were assigned to 17 strains with read counts between 5 and 60, 1.2% to 23 strains with 2 to 4 reads, and 0.9% to strains with only a single read (Figure 2b). Without prior knowledge of the number of strains in the vaccine, strain identification using megablast can be misleading, particularly if appropriate reference sequences are missing from the database. In our case, the high numbers of matched reads could lead to the incorrect conclusion that only the four isolates listed above (and/or close relatives thereof) were present in the vaccine.

### 2.4. Reference-Guided Assembly Approach on Full Genomes

A reference-guided assembly can be performed if all strains in the vaccine have been reliably identified in advance and corresponding reference sequences are available. However, in our case, an initial megablast search of the raw reads had only identified four strains, because for the fifth (Component 2/O-3039), no whole-genome sequence was available in the database. Component 2 was only later identified in a de novo assembly (see below). A stringent mapping of the raw reads against the four reference sequences identified by the megablast search produced three almost complete FMDV genomes (Component 1, 3 and 5) while a fourth (Component 4/A Malaysia 97) was split in three contigs. This demonstrates that reference-guided assembly can be used to reconstruct full genomes but only when the total number of strains present is known in advance and the corresponding reference sequences are available. If these prerequisites are not met, reference-guided assembly will give incomplete and misleading results. 

### 2.5. De Novo Assembly of Full-Genomes

The difficulty in the de novo assembly of the components of a polyvalent vaccine lies in the conserved genomic regions shared between different strains. To obtain optimal results, two assembly algorithms, GS De Novo Assembler (Newbler) and SPAdes [22], were compared. Using default settings in the de novo assembly with both methods, only fragmented genomes were obtained, indicated by many contigs being assigned to different strains. To obtain full genome sequences in the assembly, an optimal range for the assembly stringency needed to be determined as well as the data set size adjusted. For example, a highly stringent assembly combined with a high sequencing depth produced reliable contigs, but at the same time failed to provide full genome sequences. In case of the GS De Novo Assembler, very strict assembly parameters of a minimum identity of 100%, minimum overlap length of 99%, and a data set size of 20,000 reads delivered 185 contigs, with the largest contig being 1801 bp. The best assembly results were achieved with the minimum read identity set to 97% and minimum overlap length set to 99%, with a data set of 20,000 reads. With these settings, the GS De Novo Assembler returned exactly 10 contigs, where each viral genome was divided in two contigs that were separated by the poly-C region in the 5’-untranslated region. These two contigs were combined manually based on their individual BLAST results to obtain five complete FMDV genomes, including the genome of Component 2, which could not be reconstructed by reference-guided assembly due to the lack of an appropriate reference sequence. The assembly was then verified by mapping the dataset against each of the five genomes with even higher stringency and manually inspecting the read alignments, thereby also confirming the correct assembly of the two contigs with reads covering the poly-C region. The five obtained FMDV genomes have sequence identities of 91% to 100% to FMDV genomes available from the International Nucleotide Sequence Database Collaboration (INSDC; http://www.insdc.org/) (Table 1) and match the strain identification provided by the manufacturer on the label of the vaccine. The VP1 sequence of Component 2 was 100% identical to FMDV O/TAI/Ban/60 (accession no. KM243030.1), which corresponds to the vaccine strain commonly known as O-3039 [23]. A whole-genome sequence of this strain is not available from INSDC. 

The genomes themselves have pairwise sequence identities of less than 90% (Table 2).

De novo assembly with SPAdes was also attempted, but the fine-tuning of stringency that can be done by the user is limited. The “--careful” mode was applied during assembly, and different data set sizes were provided. Data sets of 5000 and 10,000 reads proved to be too small, resulting in 12 and 7 contigs, with the longest contigs amounting to 3697 and 3743 bp in length, respectively. A data set of 20,000 reads gave better results, with 3 contigs of almost 8 kb (corresponding to nearly complete genomes of Component 2, 3, and 4) and 10 contigs of 256 to 4298 bp in length. A data set of 100,000 reads returned 38 contigs, with the largest contig being 10,105 bp long, a peculiar chimera of Components 5 and 3, as well as an almost complete genome of Component 4. An input of 1 million reads produced 41 contigs, with Component 5 almost fully assembled but all other genomes again fragmented, and some additional hamster sequences. Hence, it was not possible to reliably reconstruct the genomes of the vaccine strains using SPAdes in this case.

### 2.6. Distribution of FMDV RNA

Competitive mapping of a partial dataset of 60,000 randomly selected reads against the de novo assembled genomes of the five identified strains allowed a rough estimation of the quantitative composition of the pentavalent vaccine. With highly stringent parameters, half of the reads were allocated to Component 1 (49.9%), and roughly 11% to Component 4 and 5, 8.9% to Component 3, and 5.4% to Component 2 (Table 3), respectively, resulting in only 86.1% of the reads allocated overall. When the stringency was slightly reduced, 98.2% of the dataset were allocated to the five FMDV strains (Table 3).

## 3. Discussion

The application of vaccines to prevent clinical disease and reduce viral shedding is an important component of FMD control, particularly in endemic regions. Ideally, a universal vaccine would provide full protection against all FMD strains with long-lasting immunity and without persistent infection [24,25]. However, the lack of cross-protection between different serotypes and strains is a hurdle to the successful application of current vaccines. Accordingly, a jointly published checklist for vaccine selection by the Food and Agriculture Organization of the United Nations and the World Organisation for Animal Health highlights the impact of antigenic differences between vaccine and field strains and the necessity to obtain advice on strain selection from reference laboratories [16]. This is often made difficult by a lack of disclosure of the strains used for vaccine production. Additional confusion can be caused by different manufacturers referring to different virus strains by the same name [17].

The universal approach presented here gives the opportunity to determine the full genomes of all vaccine strains, including the regions coding for the capsid proteins, in one sequencing run. The protocol does not require any modification for different vaccine components and inherits no bias from a specific amplification with possibly suboptimal primer binding. A somewhat similar approach has been described for poliovirus vaccines, where both attenuated strains and wild-type strains propagated only for inactivated vaccines, had been handled at the same production site [26], and for the quantification of low-frequency revertants in live oral poliovirus vaccines [27]. Several whole-genome sequencing approaches for FMDV have been published [28,29,30], but to our knowledge, none have been applied to inactivated vaccines. 

The BEI inactivation leads to a strong fragmentation of the RNA, but with appropriate fine-tuning of the assembly this does not hamper the analysis. In general, longer reads are favorable for the assembly process and the disambiguation of related strains. Therefore, the library containing the longer fragments (lib03330_large) was selected for sequencing, although overall, less library material was available compared to the smaller fragments. Nevertheless, several million reads were obtained with only a fraction of the library, produced with an amplification-free library preparation workflow, of which several 10,000 reads were enough to reconstruct the whole genome sequences. This was favored by the high purity of the vaccine, with a low amount of background reads as residuals from the cell line used for virus propagation. Lower quality vaccines will have an increased background of non-FMDV reads, and an inadequate sequencing depth can then lead to problems with the FMDV assembly. However, by increasing the sequencing depth, it should generally be possible to assemble at least one large contig for each virus strain. 

Local alignment searches and mapping approaches for vaccine strain identification require the corresponding reference sequences to be known. In the case presented here, only four of five genomes could be reconstructed, because for the fifth no reference sequence was available in public databases. In contrast, the open approach of de novo assembly can reconstruct sequences without prerequisites, resulting in the reconstruction of all five genomes in this case. If the strains are genetically highly similar or contain conserved regions, the assembly of short sequences is challenging and requires strict assembly parameters to distinguish between different strains and avoid the assembly of spurious chimeric constructs. In our opinion, the de novo assembly with strict parameters is to be favored over a mapping approach, even if it might result only in partial but more reliable genome sequences. With this approach, the investigator can also be certain to have identified all strains present in the vaccine product, even if the manufacturer does not provide the total number of strains. It is important to note, however, that even virus strains that are only present in trace amounts—intentionally or from contamination—will be detected. If reads are specific and unique to one assembled contig, it is highly likely that the corresponding strain was actually present in the vaccine, intentionally or otherwise. There is no unequivocal cut-off to differentiate contaminants from minor vaccine components, but we suppose that if less than 1% of all FMDV reads match a particular virus strain, it was probably not intended to be a component of the vaccine. 

After successful strain identification and genome assembly, the proportional distribution of the total viral RNA in the vaccine across the component strains can be determined. In this case, over half of the viral RNA originated from one component (O_1_ Manisa) while the other serotype O strain accounted for less than 7% of RNA. The other three strains each represented between 11% and 13% of the total RNA. This provides additional information about the vaccine composition that is not usually disclosed by the manufacturer. Due to the stringent parameters, the read assignment itself is unbiased. It is important to note, however, that the quantitative distribution of viral RNA is not necessarily the same as the distribution of viral antigen, because the RNA-to-protein ratio may vary between strains.

We compared two commonly used de novo assemblers, SPAdes and GS De Novo Assembler (Newbler), with Newbler delivering much better results. This is mostly due to its stringency parameters that can be adjusted in more detail than for SPAdes. However, other assemblers will also work, if the user can set the assembly stringency to be high enough. 

The fragmented RNA limits the maximum read length obtainable in sequencing, and we showed that the genome reconstruction is possible with Ion Torrent single-end reads, with a median read length of 277 bp after trimming. Nonetheless, sequencing on an Illumina device in a paired-end mode might provide additional benefit to the assembly.

We trust that the detailed description of our approach allows others to explore the composition of multivalent vaccines when supporting information is not available, and hope that the information gained thereby simplifies the selection of appropriate vaccines for FMD control.

## 4. Materials and Methods

### 4.1. Nucleic Acid Extraction, Library Preparation, and Sequencing

A commercial pentavalent FMD vaccine, made from purified antigens that had been inactivated by treatment with BEI and were formulated with a double oil emulsion (water in oil in water) adjuvant, was selected for the study. Total RNA was extracted from the inactivated vaccine with TRIzol LS (Thermo Fisher Scientific, Waltham, MA, USA) and RNeasy Mini spin columns (Qiagen, Hilden, Germany) with on-column DNase I digestion, as described previously [31]. In detail, 250 µL of vaccine emulsion was combined with 750 µL of TRIzol LS, mixed and incubated for 5 min at room temperature (RT), then 200 µL of chloroform was added, mixed, and incubated for 10 min, also at RT. After centrifugation for 10 min at 18,000× *g* at 4 °C, the upper aqueous phase was removed, mixed with 600 µL 75% (*v*/*v*) ethanol, and subsequently transferred in two steps onto the spin column. Binding, washing, and DNase digestion were carried out as per the manufacturer’s instructions for the RNeasy Mini Kit. Purified RNA was eluted from the column three times with 50 µL of RNase-free water for a total elution volume of 150 µL. The amount of RNA was determined by absorption at 260 nm using a NanoDrop ND1000 spectrophotometer (Thermo Fisher Scientific). Quality of extracted RNA was assessed using an RNA 6000 Pico chip on an Agilent 2100 Bioanalyzer.

For cDNA synthesis, 125 µL of RNA (=500 ng) were concentrated by adding 1.8 volumes of Agencourt RNAClean XP beads (Beckman Coulter, Brea, CA, USA) and incubating for 7 min on a shaker at 550 rpm at RT. After collecting the beads on a magnetic rack and washing twice with 700 µL 80% (*v*/*v*) ethanol, the bead pellet was air-dried and the RNA eluted in 13 µL of RNase-free water. For reverse transcription, the SuperScript IV First-Strand cDNA Synthesis System (Thermo Fisher Scientific) in combination with the NEBNext Ultra II Non-Directional RNA Second Strand Synthesis Module (New England Biolabs, Ipswich, MA, USA) was used according to the manufacturers’ instructions. The cDNA was sheared on a Covaris M220 Focused-ultrasonicator with a target size of 400 bp. Sheared DNA was concentrated by adding 1.8 volumes Agencourt AMPure XP beads (Beckman Coulter), washed twice with 80% (*v*/*v*) ethanol, eluted in 25 µL of nuclease-free water, and used for library preparation with the GeneRead DNA Library L Core kit (Qiagen). After end repair and adapter ligation, the library was purified using 1.8 volumes AMPure XP beads, followed by a size selection. In the first step, 104 µL of AMPure XP beads were diluted with 80 µL of nuclease water and added to 100 µL of purified library. After 7 min of incubation on a shaker (550 rpm at room temperature), the beads were pelleted on a magnetic rack and 250 µL of the cleared solution were combined with 30 µL of AMPure XP beads in a new tube. After a second round of incubation and pelleting, 276 µL of the cleared solution were recovered, containing only the small library molecules (size distribution ~150–350 bp, labelled “lib03330_small”) while longer molecules in the library (size distribution ~350–550 bp) remained bound to the beads. The beads were washed twice with 80% ethanol, air-dried, and the final library for sequencing (“lib03330_large”) eluted in two steps of 17 µL each. The supernatant containing the smaller fragments was purified twice with 1.2 volumes of AMPure XP beads. Quality control was performed with an Agilent High Sensitivity DNA kit and the molarity determined with a KAPA Library Quantification kit. Sequencing of lib03330_large was performed in a pooled run on an Ion Torrent S5 XL instrument with an Ion 530 Chip Kit.

### 4.2. Data Analysis

For a first impression of the proportion of FMDV reads in the vaccine library, 5000 randomly selected reads were compared against known FMDV genomes deposited in public databases (date of access: 13/09/2019) using megablast (National Center for Biotechnology Information, U.S. National Library of Medicine), with an E-value cut-off of 0.00001 and only the best hit returned for every read. For a deeper investigation into vaccine purity, the metagenomic software pipeline RIEMS [21] was used on the full dataset in its basic analysis mode. Reads identified as FMDV by megablast were sorted and counted according to their best hit.

For reference guided-assembly, the GS Reference Mapper (version 3.0; 454 Life Sciences/Roche) was used on a data set of 200,000 reads with stringency parameters of a 99% minimum overlap identity (-mi; default setting 90%) and minimum overlap length (-mL; default setting 90%). Based on the megablast results, four reference sequences (accession no. AY593823, KJ933864, AY593798, and AY593762) were used for the mapping.

For de novo assembly, the GS De Novo Assembler (Newbler version 3.0; 454 Life Sciences/Roche) was used as follows: The minimum overlap length was set to 99% while the minimum overlap identity was varied from 95% to 100% in the search for optimal results. Data set sizes ranged from 20,000 to 100,000 reads. The best-performing assembly parameters resulted in two contigs for each genome, which were manually combined according to their megablast results against the nucleotide database. For verification of the correct assembly, 500,000 reads were mapped to the assembled sequence using GS Reference Mapper with a minimum overlap length of 99% and minimum overlap identity of 98%, resulting in the final genome, and the alignments were visually inspected for correctness. For identification of the most closely related virus strain, a megablast search with default parameters was performed against the INSDC database and the hit with the highest score was selected. Pairwise nucleotide sequence identities were determined using the EMBOSS Needle Pairwise Alignment tool (version 6.3.1) with default settings. 

The 454 Genome Sequencer software package (version 3.0), including GS Reference Mapper and GS De Novo Assembler is available for download as part of the RIEMS software pipeline [21] at https://github.com/EBI-COMMUNITY/fli-RIEMS. 

In parallel, de novo assembly using SPAdes version 3.11.1 was performed with Ion Torrent single reads defined as input (--iontorrent), mismatch careful mode turned on (--careful), k-mer sizes of 21, 33, 55, 77, 99, and 127 in the mode of read error correction and subsequent assembly. Data set sizes ranged from 5000, 10,000, 20,000, 100,000 to 1 million reads. 

For proportional composition analysis, a partial dataset of 60,000 reads was competitively mapped against the five de novo assembled genomes using GS Reference Mapper with the information of uniquely mapped reads provided by the software in the RefStatus file. The highly stringent approach included a minimum overlap identity of 99% and a minimum sequence identity of 98%; the second slightly less stringent mapping approach included a minimum overlap length and a minimum sequence identity of 95% each.

## Figures and Tables

**Table 1:** Vaccine components and most closely related strains in the nucleotide database based on a megablast search of the full genome.

Vaccine Component	Closest Relative in the Nucleotide Database			
	Serotype	Strain	Accession No.	Nucleotide Identity [%]
Component 1	O	o1manisa iso87	AY593823.1	99
Component 2	O	Akesu/58	AF511039.1	91
Component 3	A	a22iraq-95 iso95	AY593762.1	99
Component 4	A	Malaysia 97	KJ933864.1	99
Component 5	Asia1	As1/Shamir/89	JF739177.1	100

**Table 2:** Pairwise sequence identities of the genomes of the component strains in percent.

	Component 1	Component 2	Component 3	Component 4	Component 5
Component 1	100.0	89.4	86.0	85.8	85.8
Component 2	89.4	100.0	85.0	84.8	84.8
Component 3	86.0	85.0	100.0	89.3	85.7
Component 4	85.8	84.8	89.3	100.0	85.7
Component 5	85.8	84.8	85.7	85.7	100.0

**Table 3:** Distribution of reads across the five identified strains.

Reference	Highly Stringent Mapping ^1^		Less Stringent Mapping ^2^	
	Unique Matching Reads *	% of All Reads	Unique Matching Reads *	% of All Reads
Component 1	29,823	49.9	32,452	54.3
Component 5	6635	11.1	7920	13.2
Component 4	6439	10.8	7917	13.2
Component 3	5320	8.9	6489	10.9
Component 2	3242	5.4	3934	6.6
Total	51,459	86.1	58,712	98.2

^1^: Highly stringent mapping parameters included a minimum overlap identity of 99% and a minimum sequence identity of 98%; ^2^: Less stringent mapping parameters included a minimum overlap identity and a minimum sequence identity of each 95%; *: Number of reads that mapped uniquely to this reference out of a total of 60,000 randomly selected reads.

## Supplementary Materials

The following are available online at https://www.mdpi.com/2076-0817/9/1/63/s1, Table S1: Summarized result of a megablast search of a partial dataset of 5000 randomly selected reads against known FMDV genomes.

## References

[1] Klein J. (2009). Understanding the molecular epidemiology of foot-and-mouth-disease virus. Infect. Genet. Evol..

[2] Alexandersen S., Mowat N. (2005). Foot-and-mouth disease: Host range and pathogenesis. Curr. Top. Microbiol. Immunol..

[3] Chowdhury S.M.Z.H., Rahman M.F., Rahman M.B., Rahman M.M. (1993). Foot and mouth disease and its effects on morbidity, mortality, milk yield and draft power in Bangladesh. Asian-Aust. J. Anim. Sci..

[4] FAO (2016). Foot-and-Mouth Disease Situation—Monthly Report September 2016.

[5] Stenfeldt C., Eschbaumer M., Rekant S.I., Pacheco J.M., Smoliga G.R., Hartwig E.J., Rodriguez L.L., Arzt J. (2016). The Foot-and-Mouth Disease Carrier State Divergence in Cattle. J. Virol..

[6] Aphis, USDA (2018). Factsheet: Foot and Mouth Disease and Vaccine Use.

[7] Cox S.J., Voyce C., Parida S., Reid S.M., Hamblin P.A., Hutchings G., Paton D.J., Barnett P.V. (2006). Effect of emergency FMD vaccine antigen payload on protection, sub-clinical infection and persistence following direct contact challenge of cattle. Vaccine.

[8] Parida S. (2009). Vaccination against foot-and-mouth disease virus: Strategies and effectiveness. Exp. Rev. Vaccines.

[9] Gao Y., Sun S.Q., Guo H.C. (2016). Biological function of Foot-and-mouth disease virus non-structural proteins and non-coding elements. Virol. J..

[10] Domingo E., Domingo E. (2016). Chapter 2—Molecular basis of genetic variation of viruses: Error-prone replication. Virus as Populations.

[11] Domingo E., Baranowski E., Escarmís C., Sobrino F. (2002). Foot-and-mouth disease virus. Comp. Immunol Microbiol. Infect. Dis..

[12] Fernandez-Sainz I., Gavitt T.D., Koster M., Ramirez-Medina E., Rodriguez Y.Y., Wu P., Silbart L.K., de Los Santos T., Szczepanek S.M. (2019). The VP1 G-H loop hypervariable epitope contributes to protective immunity against Foot and Mouth Disease Virus in swine. Vaccine.

[13] Kitching R.P., Knowles N.J., Samuel A.R., Donaldson A.I. (1989). Development of foot-and-mouth disease virus strain characterisation—A review. Trop. Anim. Health Prod..

[14] Singh R.K., Sharma G.K., Mahajan S., Dhama K., Basagoudanavar S.H., Hosamani M., Sreenivasa B.P., Chaicumpa W., Gupta V.K., Sanyal A. (2019). Foot-and-Mouth Disease Virus: Immunobiology, Advances in Vaccines and Vaccination Strategies Addressing Vaccine Failures-An Indian Perspective. Vaccines.

[15] Ullah H., Siddique M.A., Al Amin M., Das B.C., Sultana M., Hossain M.A. (2015). Re-emergence of circulatory foot-and-mouth disease virus serotypes Asia1 in Bangladesh and VP1 protein heterogeneity with vaccine strain IND 63/72. Lett. Appl. Microbiol..

[16] Ferrari G., Paton D., Duffy S., Bartels C., Knight-Jones T. (2016). Foot and Mouth Disease Vaccination and Post-Vaccination Monitoring.

[17] Paton D.J., Valarcher J.F., Bergmann I., Matlho O.G., Zakharov V.M., Palma E.L., Thomson G.R. (2005). Selection of foot and mouth disease vaccine strains—A review. Rev. Sci. Tech..

[18] Je S.H., Kwon T., Yoo S.J., Lee D.U., Seo S.W., Byun J.J., Shin J.Y., Lyoo Y.S. (2018). Genetic identification and serological evaluation of commercial inactivated foot-and-mouth disease virus vaccine in pigs. Clin. Exp. Vaccine Res..

[19] Amaral-Doel C.M., Owen N.E., Ferris N.P., Kitching R.P., Doel T.R. (1993). Detection of foot-and-mouth disease viral sequences in clinical specimens and ethyleneimine-inactivated preparations by the polymerase chain reaction. Vaccine.

[20] Ludi A.B., Horton D.L., Li Y., Mahapatra M., King D.P., Knowles N.J., Russell C.A., Paton D.J., Wood J.L., Smith D.J. (2014). Antigenic variation of foot-and-mouth disease virus serotype A. J. Gen. Virol..

[21] Scheuch M., Höper D., Beer M. (2015). RIEMS: A software pipeline for sensitive and comprehensive taxonomic classification of reads from metagenomics datasets. BMC Bioinform..

[22] Bankevich A., Nurk S., Antipov D., Gurevich A.A., Dvorkin M., Kulikov A.S., Lesin V.M., Nikolenko S.I., Pham S., Prjibelski A.D. (2012). SPAdes: A new genome assembly algorithm and its applications to single-cell sequencing. J. Comput. Biol..

[23] Park J.H., Tark D., Lee K.N., Chun J.E., Lee H.S., Ko Y.J., Kye S.J., Kim Y.J., Oem J.K., Ryoo S. (2018). Control of type O foot-and-mouth disease by vaccination in Korea, 2014-2015. J. Vet. Sci..

[24] Mahapatra M., Parida S. (2018). Foot and mouth disease vaccine strain selection: Current approaches and future perspectives. Exp. Rev. Vaccines.

[25] Park J.H. (2013). Requirements for improved vaccines against foot-and-mouth disease epidemics. Clin. Exp. Vaccine Res..

[26] Mee E.T., Minor P.D., Martin J. (2015). High resolution identity testing of inactivated poliovirus vaccines. Vaccine.

[27] Sarcey E., Serres A., Tindy F., Chareyre A., Ng S., Nicolas M., Vetter E., Bonnevay T., Abachin E., Mallet L. (2017). Quantifying low-frequency revertants in oral poliovirus vaccine using next generation sequencing. J. Virol. Methods.

[28] Hansen S., Dill V., Shalaby M.A., Eschbaumer M., Bohlken-Fascher S., Hoffmann B., Czerny C.P., Abd El Wahed A. (2019). Serotyping of foot-and-mouth disease virus using oxford nanopore sequencing. J. Virol. Methods.

[29] Logan G., Freimanis G.L., King D.J., Valdazo-Gonzalez B., Bachanek-Bankowska K., Sanderson N.D., Knowles N.J., King D.P., Cottam E.M. (2014). A universal protocol to generate consensus level genome sequences for foot-and-mouth disease virus and other positive-sense polyadenylated RNA viruses using the Illumina MiSeq. BMC Genom..

[30] Moser L.A., Ramirez-Carvajal L., Puri V., Pauszek S.J., Matthews K., Dilley K.A., Mullan C., McGraw J., Khayat M., Beeri K. (2016). A Universal Next-Generation Sequencing Protocol To Generate Noninfectious Barcoded cDNA Libraries from High-Containment RNA Viruses. mSystems.

[31] Wylezich C., Papa A., Beer M., Höper D. (2018). A Versatile Sample Processing Workflow for Metagenomic Pathogen Detection. Sci. Rep..

