# Supplementary material for: High-Resolution Composition Analysis of an Inactivated Polyvalent Foot-and-Mouth Disease Vaccine
Source: Pathogens. 2020 Jan 16;9(1):63. doi: 10.3390/pathogens9010063 (PMC7168581; doi:10.3390/pathogens9010063)
Supplement: Supplementary file 1 [file pathogens-09-00063-s001.pdf]

Supplementary Material to

"Unbiased high-resolution composition analysis of an inactivated polyvalent foot-and-mouth disease vaccine"

by L.F. Forth, D. Höper, M.Beer and M. Eschbaumer

Supplementary Table S1: Summarized result of a megablast search of a partial dataset of 5000 randomly selected reads against known FMDV genomes. The corresponding strain/isolate name is provided if ≥5 reads matched, for others only the accession numbers are listed.

Assigned reads	Accession no.	Strain/isolate name
2722	AY593823.1	Foot-and-mouth disease virus O isolate o1manisa iso87, complete genome
673	KJ933864.1	Foot-and-mouth disease virus - type A strain Malaysia 97, complete genome
615	AY593798.1	Foot-and-mouth disease virus Asia 1 isolate asia1leb-89 iso89, complete genome
560	AY593762.1	Foot-and-mouth disease virus A isolate a22iraq-95 iso95, complete genome
53	MG372731.1	Foot-and-mouth disease virus - type Asia 1 isolate TBD, complete genome
38	AF511039.1	Foot-and-mouth disease virus - type O strain Akesu/58, complete genome
31	AY359854.1	Foot-and-mouth disease virus O strain OMIII, complete genome
28	KY072818.1	Foot-and-mouth disease virus - type O isolate YNTBa, complete genome
23	DQ533483.2	Foot-and-mouth disease virus - type Asia 1 strain ZB/CHA/58(att), complete genome
22	DQ478936.1	Foot-and-mouth disease virus - type O, complete genome
21	AY390432.1	Foot-and-mouth disease virus Asia1 strain YNBS/58, complete genome
14	JF739177.1	Foot-and-mouth disease virus - type Asia 1 isolate As1/Shamir/89, complete genome
9	AY687333.1	Foot-and-mouth disease virus - type Asia 1 isolate IND 321/01, complete genome
8	DQ989305.1	Foot-and-mouth disease virus - type Asia 1 isolate IND 116-90, complete genome
8	DQ989309.1	Foot-and-mouth disease virus - type Asia 1 isolate IND 82-96, complete genome
6	AY593821.1	Foot-and-mouth disease virus O isolate o1caseros iso35, complete genome
6	KJ206909.1	Foot-and-mouth disease virus - type O isolate LIB/2/2013 polyprotein gene, complete cds
5	AY593752.1	Foot-and-mouth disease virus A isolate a12valle 119 iso20, complete genome
5	AY593791.1	Foot-and-mouth disease virus A isolate airan iso105, complete genome
5	MH053313.1	Foot-and-mouth disease virus - type O isolate ETH/2/2006, complete genome
5	MH053318.1	Foot-and-mouth disease virus - type O isolate UGA/3/2002, complete genome

+ 102 further reads matched to 66 strains:
AY593796.1, HQ832576.1, HQ832585.1, AF308157.1, AY593795.1, AY593813.1, AY593828.1, HM854021.1, HM854022.1, KY322675.1, AB079061.1, AY593777.1, AY593783.1, AY593808.1, AY593811.1, DQ989313.1, EF117837.1, GU384682.1, HQ632769.1, KJ825807.1, KM268896.1, KY234501.1, MF461724.1, AF026168.2, AJ320488.1, AJ539138.1 ,AY593763.1, AY593765.1, AY593772.1, AY593793.1, AY593797.1, AY593803.1, AY593806.1, AY593807.1, AY593809.1, AY593824.1, DQ989306.1, DQ989315.1, DQ989316.1, DQ989319.1, FJ175663.1, FJ906802.1, HQ631363.1, HQ632768.1, HQ632770.1, HQ632772.1, HQ832589.1, JN998086.1, KC462884.1, KF112879.1, KF112884.1, KF694731.1, KJ754939.1, KJ825801.1, KJ825803.1, KM257065.1, KM268898.1, KY446902.1, LC149617.1, LC438822.1, MH053305.1, MH053307.1, MH053309.1, MH053312.1, MH053316.1, MK071699.1

Pathogens **2020**, *9*, 63; doi:10.3390/pathogens9010063
